# Navigating the Landscape of Cancer From Ancient Times to Modern Challenges: A Narrative Review

**DOI:** 10.7759/cureus.65230

**Published:** 2024-07-23

**Authors:** Jitendra Bhawalkar, Akash Nagar, Hetal Rathod, Prerna Verma

**Affiliations:** 1 Community Medicine, Dr. D. Y. Patil Medical College Hospital and Research Centre, Dr. D. Y. Patil Vidyapeeth (Deemed to be University), Pune, IND

**Keywords:** early detection, risk factors, treatment barriers, screening practices, cancer burden, cancer history

## Abstract

Cancer, a pervasive and multifaceted disease, has afflicted humanity since ancient times, as evidenced by early references in the Edwin Smith Papyrus and the Ebers Papyrus. Over centuries, our understanding and treatment of cancer have evolved significantly, transitioning from rudimentary remedies to advanced modalities like chemotherapy, radiation therapy, and precision medicine. Despite these advancements, cancer remains a major global health challenge. As of 2022, nearly 20 million new cancer cases and 10 million cancer-related deaths were reported worldwide. In India, the situation is particularly dire, with over 1.41 million new cases and more than 916,827 deaths in 2022, exacerbated by socioeconomic disparities, cultural stigmas, and healthcare barriers. This review traces the historical evolution of cancer treatment from ancient civilizations to modern times, highlighting key medical milestones and breakthroughs. It examines the global and Indian cancer burden, emphasizing the critical barriers to early diagnosis and effective treatment. These barriers include health system deficiencies, socioeconomic challenges, delayed diagnosis, low health literacy, and inadequate screening programs. The review was conducted through a comprehensive literature search using databases such as PubMed, Google Scholar, Journal Storage (JSTOR), and various other sources focusing on historical texts, epidemiological studies, and current medical research. The search aimed to gather a broad spectrum of perspectives and evidence to provide a well-rounded understanding of cancer's historical journey and current landscape.

## Introduction and background

Cancer, a term that invokes fear and urgency, has plagued humanity for millennia, evolving into a multifaceted challenge that transcends medical, social, and economic dimensions. The historical tapestry of cancer reveals our persistent struggle against a disease that has been documented since ancient civilizations, with references in the Edwin Smith Papyrus and Ebers Papyrus dating back to 3000 BC and 1500 BC, respectively [1]. Over centuries, medical understanding and treatment of cancer have undergone profound transformations, from ancient herbal remedies and surgical techniques to the advent of chemotherapy, radiation therapy, and precision medicine.

The relentless pursuit of cancer cures and management strategies has led to significant breakthroughs, yet the global burden of cancer remains staggering. Nearly 20 million new cases of cancer and almost 10 million cancer-related deaths occurred globally in 2022 [2]. Cancer is a major cause of illness and death, according to epidemiological patterns, and its incidence and survival rates vary depending on a person's lifestyle, genetics, availability of early detection and treatment, and the healthcare system [3].

In India, the cancer landscape is particularly challenging. The nation faces a significant disease burden in 2022, with over 1.41 million instances of disease and over 916,827 fatalities. This burden is exacerbated by socioeconomic inequities, cultural stigmas, and obstacles in the way of the healthcare system. The situation is worsened by the prevalence of risk factors such as diabetes, alcoholism, and tobacco use, as well as by delayed diagnosis and treatment [4-7].

This review delves into the rich history of cancer, tracing its origins and the evolution of medical practices aimed at combating the disease. It also examines the current global and Indian cancer burden, highlighting the critical barriers to timely diagnosis and effective treatment. By exploring the risk factors, delays in treatment, and socio-economic impacts, this review highlights the need for continued research, policy interventions, and public health initiatives. This descriptive review used various search strategies to identify publications providing data on cancer from the earliest available records. A variety of keywords (cancer, history of cancer, barriers to diagnosis, risk factors) were used for searching the databases, including PubMed, Google Scholar, PubMed Central (PMC), and the archives of digital libraries. We also searched online publications and databases from different wings of the Government of India (GOI), the World Health Organization (WHO), the International Agency for Research on Cancer, and the Global Cancer Observatory to gather disease burden and cancer statistics both in India and worldwide. Through a historical lens and contemporary perspective, this review aims to provide a comprehensive understanding of cancer's journey and the ongoing efforts to mitigate its impact, offering insights into future directions in the fight against this formidable disease.

## Review

History of cancer: “The history of cancer is as old as the history of mankind”

Global Cancer History

The history of cancer spans thousands of years, with ancient civilizations documenting early encounters with the disease and attempting various remedies. The Edwin Smith Papyrus, dating back to 3000 BC, contains one of the earliest known references to breast cancer, acknowledging the presence of large breast tumors as indicators of serious illness. Similarly, the Ebers Papyrus from approximately 1500 BC mentions potential malignancies affecting various organs, including the skin, uterus, stomach, and rectum, as well as soft tissue tumors like lipomas [1].

In their quest to combat cancer, ancient civilizations employed diverse treatment methods. The Egyptians, for instance, introduced arsenic paste known as "Egyptian ointment," which remained in use until the 19^th^ century. Meanwhile, the Chinese and Indians favored herbal remedies, occasionally resorting to solutions and pastes containing copper, iron, mercury, and sulfur, among other substances, for severe cases [8].

The Greeks, led by figures like Hippocrates, adopted a rational approach to medicine, attributing cancer to natural factors rather than superstitions. They believed that imbalances in bodily fluids, such as blood, mucus, and bile, particularly in old age, could trigger cancer. Hippocrates metaphorically likened cancerous growths to the movement of a crab, thus coining terms like carcinoma for malignant tumors and cancer for ulcerated malignant tumors. Additionally, he distinguished between different types of tumors, such as scirrhous (hard tumor) and carcinoma, considering them tumors with uncertain malignant potential. Hippocrates' discoveries led to the classification of tumors into deep and surface carcinomas, each requiring a different course of therapy. When it came to treating superficial lesions, creams and cauterization were frequently employed, whereas deep tumors were either surgically removed or considered incurable [9].

Continuing the Hippocratic tradition, Aulus Celsus likened cancer to a hermit that has become firmly attached to its surroundings. In "De Medicina," he listed numerous external malignancies and identified several that affected interior organs like the stomach, spleen, liver, and intestine. Aggressive and rapid surgery as a cancer therapy was promoted by Celsus. Celsus advocated for rapid and aggressive surgical treatment of cancer. He also identified the tendency of advanced breast cancer to recur in the armpits and recognized the danger of its spreading to distant organs, emphasizing the need for careful management of such cases [10].

In ancient times, Aretaeus, a physician from Alexandria, Egypt, provided detailed accounts of uterine cancer, identifying distinct types characterized by different textures and ulceration patterns. Aretaeus considered both types of uterine cancer chronic and fatal, with the ulcerated form being particularly severe and incurable [11]. He regarded uterine bleeding and enlargement as irreversible conditions.

During his practice in Rome, Claudius Galen associated cancer with thick black bile for ulcerated and incurable cancer, while non-ulcerated and treatable cancer was linked to thin yellow bile. Despite his role as a gladiator surgeon, Galen opposed surgical intervention for cancer, aligning with prevailing Roman beliefs. Instead, he suggested the use of purgatives to reduce the buildup of black bile [12]. For many years, Galen's theories, which drew from Christian theology as well as Constantinople and Arabic teachings, hindered the advancement of knowledge regarding cancer. Following the fall of the Western Roman Empire, medical knowledge centers such as Istanbul and Iraq formed, attracting contributions from physicians such as Rhazes and Oribasius of Baghdad. Oribasius noted that cancers often did not cause pain and appeared less red compared to inflammatory lesions [13]. Rhazes played a critical role in disseminating the medical works of Hippocrates and Galen in the Arabic world and made significant contributions to surgery [14].

Avicenna, a Persian scholar and physician, devised a technique for polypectomy using a wire loop that was tightened gradually over several days until the tumor detached. In France, Lanfranc and Henri de Mondeville contributed to medical research, with Lanfranc providing the first explanation on how to differentiate between benign breast tumors and cancer [15], while Mondeville openly disputed Galen's longstanding theories [16].

John Arderne considered the first proctologist, advocated for local excision as the primary treatment for cancer based on his observations of rectal cancer patients [17]. Additionally, chemicals have been used for therapeutic purposes since the 16^th^ century, with Paracelsus introducing various substances for internal remedies, albeit warning about their toxicity [18].

The 17^th^ and 18^th^ centuries witnessed advancements in pathology and surgery, with pioneers like Marco Aurelio Severino making significant contributions to tumor classification and treatment [19]. Meanwhile, the 18^th^ and 19^th^ centuries saw the introduction of radical surgical procedures and the discoveries of X-rays, revolutionizing cancer diagnosis and treatment [20].

From the mid-20^th^ century on, breakthroughs in anticancer drugs, chemotherapy, and radiation therapy transformed cancer treatment. Research intensified with the identification of carcinogens like tobacco products, leading to groundbreaking discoveries by researchers like Richard Doll and Bradford Hill [21]. The elucidation of DNA structure in 1953 by Watson and Crick provided crucial insights into oncogenesis, paving the way for novel perspectives on cancer prevention and diagnosis [22].

Between 1970 and 1995, significant advancements in cancer research and treatment occurred, including the introduction of multimodality therapy and targeted therapies like monoclonal antibodies and immunotherapy. Multidisciplinary care involving various specialists became emphasized for optimal patient outcomes [23].

In the following decades, from 1995 to 2024, there were remarkable strides in cancer research and treatment. The development of targeted therapies such as anastrozole and trastuzumab, along with breakthroughs in immunotherapy like ipilimumab and CAR T-cell therapies, marked significant milestones [24]. Landmark clinical trials, such as the Breast Cancer Prevention Trial (STEMVAC) and the Trial Assigning Individualized Options for Treatment (TAILORx) trial, reshaped treatment approaches [25, 26]. Initiatives like the Cancer Moonshot and Paediatric MATCH accelerated research efforts, while advances in genomic profiling and precision medicine led to personalized cancer care [27, 28].

**Figure 1:**
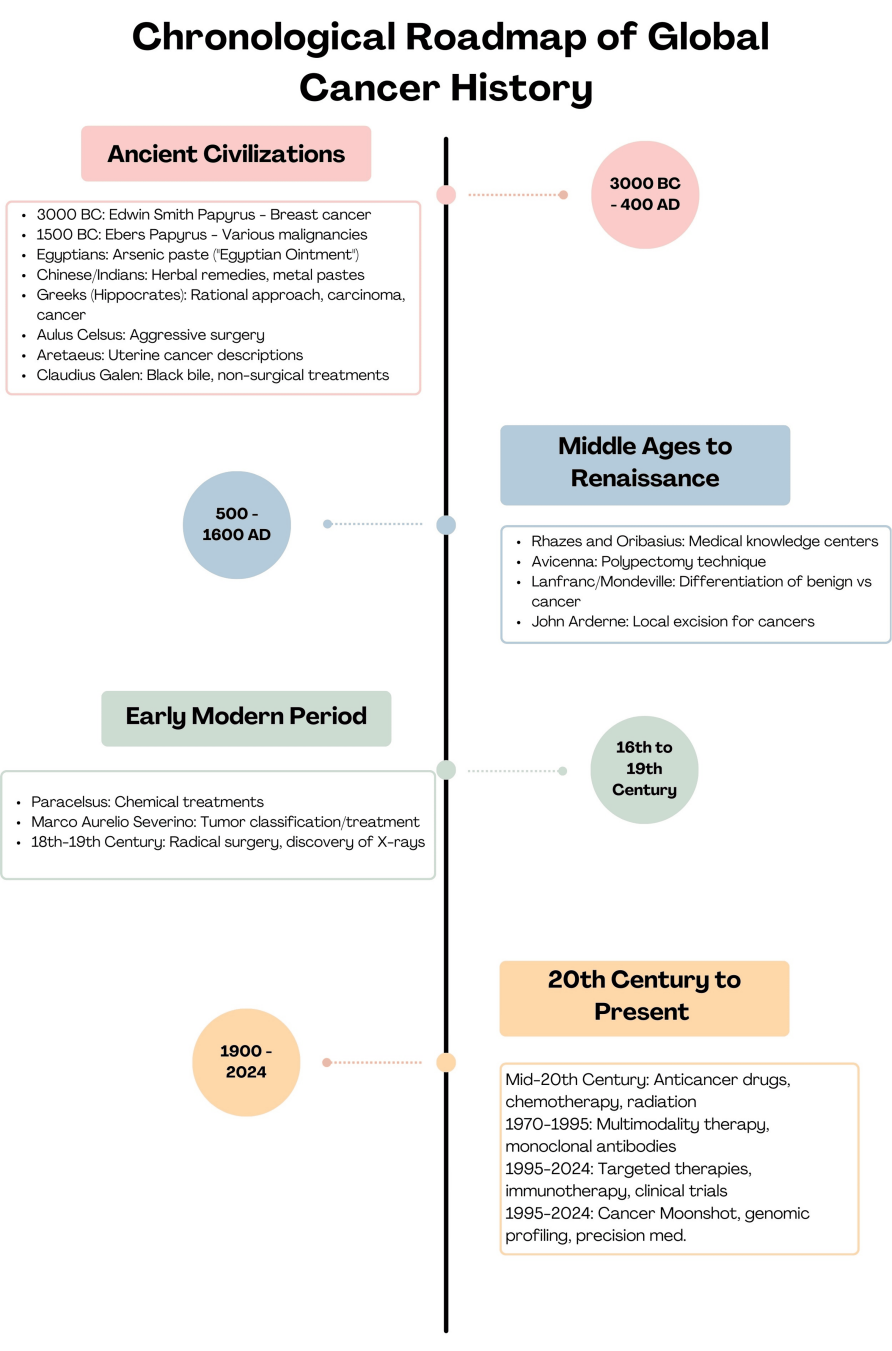
Roadmap of global cancer history This image has been created by the authors. Source: [1-28]

Throughout history, the fight against cancer has been characterized by relentless pursuit, innovative discoveries, and collaborative efforts across cultures and disciplines. From ancient civilizations' herbal remedies to modern genomic profiling and targeted therapies, the understanding and treatment of cancer have evolved significantly (Figure 1). Each milestone represents a step forward in the ongoing battle against this complex disease, offering hope for improved outcomes and, ultimately, a cure.

Cancer History in India

The history of cancer in India dates back to ancient times, with Ayurvedic and Siddha manuscripts referencing cancer-like diseases such as Arvada, Granthi, and Gulma. Medieval Indian literature seldom mentioned cancer, with case reports starting to appear in the 17^th^ century. A landmark study between 1917 and 1932 confirmed cancer as a significant cause of death among middle-aged and elderly Indians [29]. The 19^th^ century marked the onset of cancer diagnosis in India, with numerous case reports and studies documenting various types of cancers and their prevalence [30].

The 20^th^ century was pivotal in addressing India's cancer burden, with the establishment of the Indian Medical Service and the creation of medical colleges, which significantly improved cancer diagnosis and management. Landmark studies by Nath and Grewal provided valuable insights into cancer prevalence and mortality rates [31]. Post-independence, the development of comprehensive cancer care facilities, such as the Tata Memorial Hospital, and initiatives to enhance cancer diagnostics and treatment marked significant progress.

**Figure 2:**
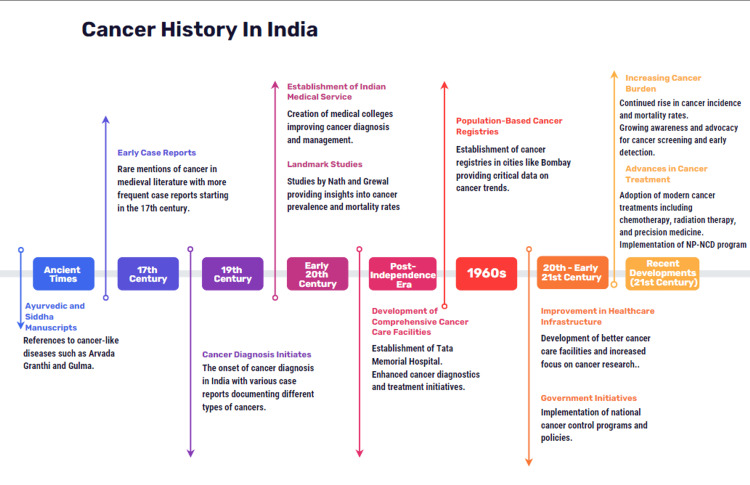
Roadmap of cancer history in India This image has been created by the authors. Source: [29-33]

In 1963, population-based cancer registries were established in cities like Bombay (Figure 2), offering critical data on cancer trends over the years [32]. The demographic transition in India has influenced the cancer burden, with significant variations between states. Lessons from India's experience with cancer provide valuable insights for developing countries facing similar challenges, emphasizing the importance of understanding evolving epidemiological patterns, improving healthcare infrastructure, and addressing the financial implications of cancer care [33].

Burden of cancer

Global Burden

In 2019, the World Health Organization (WHO) reported that cancer was the leading or second leading cause of death before age 70 in 112 out of 183 countries and ranked third or fourth in 23 additional countries [34]. By 2020, global cancer incidence rates were 19% higher in men compared to women, with men also experiencing a significantly higher mortality rate (120.8 per 100,000) compared to 84.2 per 100,000 for women, a 43% difference [35]. In 2022, there were approximately 19.97 million new cancer cases and 9.74 million cancer deaths worldwide. Lung cancer was the most commonly diagnosed cancer (12.4%), followed by breast (11.5%), colorectal (9.6%), prostate (7.3%), and stomach cancers (4.8%). It also accounted for the most deaths (18.7%), followed by colorectal (9.3%) and liver cancers (7.8%). Breast cancer had the highest five-year prevalence rate at 15.3 per 100,000, followed by colorectal and prostate cancers at 10.8 per 100,000 each.

**Figure 3:**
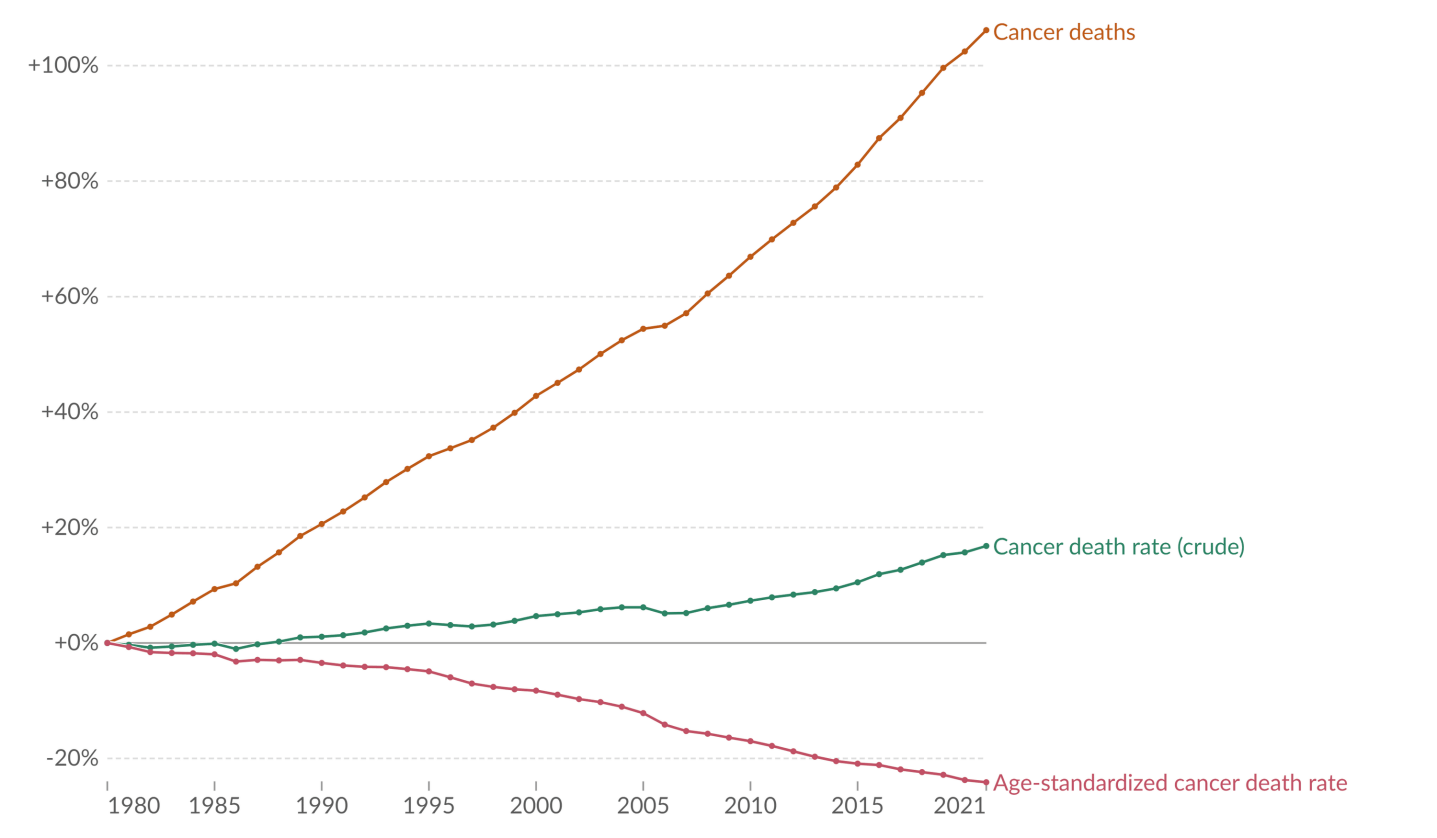
Change in three measures of cancer mortality (World, 1980 to 2021) Source: Max Roser and Hannah Ritchie (2015) - “Cancer” Published online at OurWorldInData.org. Retrieved from: 'https://ourworldindata.org/cancer' (Online Resource) [37]

Around 20% of people will develop cancer in their lifetime, with one in nine men and one in 12 women dying from it. For men, the most commonly diagnosed cancers were lung (15.2%), prostate (14.2%), colorectal (10.4%), and stomach (6.1%). For women, the most common were breast (23.8%), lung (9.4%), colorectal (8.9%), and cervical (6.9%). The leading causes of cancer deaths for men were lung (22.7%), liver (9.6%), colorectal (9.2%), and stomach (7.9%), while for women, they were breast (15.4%), lung (13.5%), colorectal (9.4%), and cervical (8.1%). Cervical cancer remains a significant global health issue, being the eighth most common cancer and the ninth leading cause of cancer deaths, with 662,301 new cases and 348,874 deaths in 2022, predominantly affecting women in 25 countries, many in sub-Saharan Africa. The International Agency for Research on Cancer (IARC) noted that 10 types of cancer accounted for about two-thirds of new cases and deaths in 2022. Cancer has demonstrated a rising trend over the years (Figure 3); further, the WHO estimates that global cancer diagnoses will rise to approximately 35 million by 2050, a 77% increase from 2022 [36,37].

Cancer Burden In India

**Figure 4:**
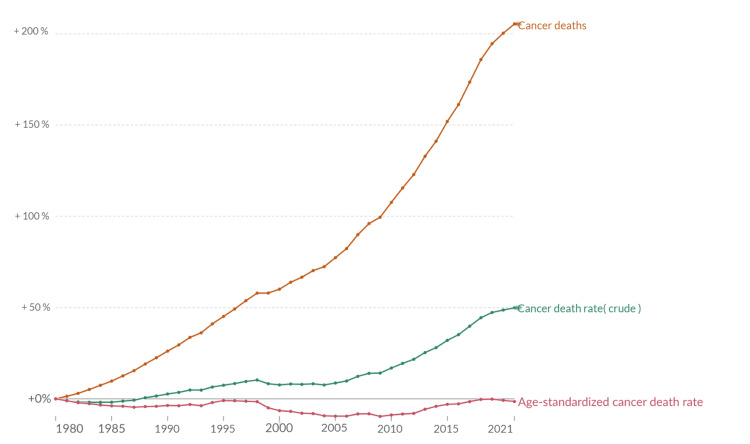
Change in three measures of cancer mortality (India, 1980 to 2021) Source: Max Roser and Hannah Ritchie (2015) - “Cancer” Published online at OurWorldInData.org. Retrieved from: 'https://ourworldindata.org/cancer' (Online Resource) [37]

In 2022, India reported over 1.41 million new cancer cases and more than 916,827 deaths, according to WHO estimates. Among men, the most common cancers were lip, oral cavity (15.6%), and lung (8.5%). For women, breast and cervical cancers were predominant, accounting for 27% and 18% of new cases, respectively. The most prevalent cancers were breast cancer (16.2 per 100,000), lip and oral cavity cancer (11.4 per 100,000), and cervical cancer (10.4 per 100,000) [36]. The Global Cancer Observatory estimated that breast, oral, and cervical cancers constituted 32% of new cancer cases in India, with a slightly higher incidence in women (722,138 cases) than in men (691,178 cases). The risk of developing cancer before age 75 was 10.6%, with a 7.2% risk of dying from it [35]. The Indian Council of Medical Research-National Cancer Registry Programme (ICMR-NCRP) projected an increase in cancer cases from 1.46 million in 2022 to 1.57 million in 2025, reaching 2.4 million by 2045, a 73.8% rise from 2020 levels [38]. These data align with the ongoing upward trajectory of cancer mortality rates observed in India over recent years (Figure 4) [37].

Current challenges

Barriers to Cancer Diagnosis and Treatment

Healthcare and behavioral factors: Afaya et al. (2022) conducted a systematic review of 26 studies from 10 Asian countries, revealing significant health system barriers impacting the timely detection and treatment of breast cancer. These obstacles fell into five categories: provision of health services, healthcare workforce, healthcare financing, health information systems, and access to essential medicines and technology. The most prevalent issue was poor healthcare delivery quality, followed by a shortage of physicians, with health information systems being the least significant obstacle [39]. Dey et al. conducted a qualitative study in Delhi, India, with 20 focus groups involving diverse women. They identified behavioral barriers to early breast cancer detection, such as shyness, fear, and postponement. Many women erroneously believed that pain was an initial breast cancer symptom. Financial constraints also delayed treatment access and social stigma associating breast problems with poor character led to women hiding symptoms [40].

Screening, "screaming for screening": Poor awareness may result in low uptake of screening modalities and delays in diagnosis. According to National Family Health Survey (NFHS-5) data, only 1.9% of women in India have ever undergone cervical cancer screening, with state-level variations ranging from 9.8% in Tamil Nadu to as low as 0.2% in West Bengal, Assam, and Gujrat. In about 50% of the states or Union Territories, less than 1% of women have participated in cervical cancer screening. Just 0.9% of women in the country have ever received a breast exam to check for breast cancer. With 5.6% of women screened for breast cancer, Tamil Nadu had the highest rate, while Chandigarh had no recorded screenings. Women's participation in oral cancer screening was likewise 0.9%, with the lowest rates in West Bengal, Chhattisgarh, Jharkhand, Assam, Rajasthan, Gujarat, and Ladakh (0.2%) and the highest in the Andaman and Nicobar Islands (10.1%). For men, the participation rate in oral cancer screening was 1.2%, with Andhra Pradesh having the highest rate (6.3%) and Lakshadweep and Ladakh having the lowest (0%) [41].

A study conducted in Nigeria mentioned that the screening challenges include information scarcity (40.6%), inconvenient screening times (37.1%), and screening anxiety (32.0%). Despite high levels of knowledge about cervical cancer among participants, screening practices remained low [42].

In their systematic analysis, Goodwin et al. found that although dread of the screening process itself can be a barrier, anticipatory anxiety about a cancer diagnosis may boost screening participation. [43].

Distance, "far from fate: The study "Modelling the Structural Relationships between Travel Distance, Built Environment, and Cancer Outcomes" found that longer travel distances to transit hospitals result in fewer radiotherapy visits for patients, impacting their treatment frequency [44].

Ambroggi et al.'s systematic review noted that greater travel requirements correlate with advanced disease at diagnosis, inappropriate treatment, poorer prognosis, and reduced quality of life [45].

Another study indicated that breast cancer patients tend to have longer delays in diagnosis when facing greater travel distances (≥5 km) [46].

Risk factors

Family History

The occurrence of breast cancer in one or more close blood relatives suggests a familial pattern. Some families experience more breast cancer cases than would be expected by chance. It can be challenging to discern whether a family’s cancer history is due to coincidence, shared lifestyle factors, inherited genes, or a combination of these elements. A study analyzed data from over 113,000 UK women to assess breast cancer risk using a family history score (FHS) that accounts for family size, age structure, and national cancer rates. Results showed that higher FHS significantly increased breast cancer risk, with a 3.5-fold rise in the highest FHS group, outperforming traditional methods that only consider the number of affected relatives. The optimal risk model combined FHS with the age at diagnosis of relatives, offering superior risk discrimination [47].

Addiction: “Break the Habit, The Best Break of Your Life”

A collaborative study by researchers from IARC, Queen Mary University of London, and King’s College London identified tobacco smoking as the leading cause of preventable cancer deaths in Brazil, Russia, India, China, South Africa, the UK, and the USA. It accounted for 1.3 million deaths, or over two-thirds of the preventable cancer deaths linked to tobacco, alcohol, obesity, and human papillomavirus (HPV) infections [48]. In 2020, alcohol consumption was responsible for approximately 741,300 new cancer cases globally, or 4.1% of all cases, with males representing 76.7%. The cancers most associated with alcohol were esophageal (189,700 cases), liver (154,700 cases), and breast cancer (98,300 cases) [49]. The IARC classifies tobacco as a Group I carcinogen, with India having the highest number of oral cancer cases globally. In India, 80%-90% of oral cancer cases are tobacco-related [50].

Age: "Coming Home Early’

Trends indicate an increasing incidence of certain cancers among younger age groups. A study by the Washington University School of Medicine found a steady rise in breast cancer diagnoses in women under 50 over the past two decades, with recent significant increases [51]. Murthy et al., in their study, using WHO data from 2000-2019, analyzed premature mortality trends for all cancers and 13 specific types across 183 countries. Findings showed that while 75% of countries have declining premature mortality rates, only 4% are on track to meet Sustainable Development Goals (SDG) 3.4 targets. High-income countries see greater reductions, especially in cancers with early detection strategies like breast and colorectal cancer, compared to low-income countries. Cervical cancer, with effective prevention programs, shows significant declines in both high- and low-income countries [52]. Additionally, the Health of the Nation Report 2024 by Apollo Hospital Group predicts a doubling of colon cancer cases in individuals under 50 in the next decade [53].

Diabetes: "Sweet Tooth Turns Bitter"

In 2019, 463 million individuals (9.3% of the world's population) had diabetes. By 2030 and 2045, respectively, this proportion is predicted to increase to 578 million (10.2%) and 700 million (10.9%). People with type 2 diabetes mellitus had higher cancer mortality rates, with pancreatic cancer mortality rising by 30%-40% and liver cancer mortality rising by 2.5 times and 30%, 15%-30%, and 20%-50% increases in endometrial, breast, and colorectal cancers, respectively [54].

Although the biological mechanisms linking diabetes and cancer are not fully understood, preliminary evidence suggests a connection. Metformin may reduce cancer risk, while exogenous insulin might increase it. Type 2 diabetes mellitus is notably associated with higher risks of liver, pancreatic, endometrial, colorectal, and breast cancers, but a lower risk of prostate cancer. Type 1 diabetes mellitus also increases cancer risk, especially for cervical and stomach cancers, with shared risk factors like aging, obesity, diet, and inactivity potentially contributing to these associations [55].

An umbrella review by Tsilidis et al. examined the links between type 2 diabetes mellitus and cancer risk at 20 sites and cancer mortality at seven sites, finding robust evidence without bias in only a few studies [56].

Food-Related Carcinogens: "What’s On Your Plate!"

A study conducted by Vailidandi et al. in Hyderabad, Telangana, India, investigated aflatoxin (AF) contamination in rice samples collected from local markets and also estimated the average liver cancer risk associated with rice consumption. The study revealed that 54% of the samples were contaminated with AF B1 and 34% with AF B2, with concentrations ranging from 0-20.35 µg/kg and 0-1.54 µg/kg, respectively. Notably, three samples exceeded the Food Safety and Standards Authority of India's (FSSAI) total AF acceptable limit of 15 µg/kg. Furthermore, the results showed that the average risk of liver cancer among this rice-consuming population was 0.27, 0.28, and 0.40 hepatocellular carcinoma (HCC) cases per year per 100,000 individuals for adults, adolescents, and children, respectively [57].

Banerjee et al. explored the potential links between dietary patterns, lifestyle habits, and gallbladder carcinoma (GBC) risk. A case-control study examined 56 GBC patients and 56 matched controls, revealing significant associations between certain dietary elements and GBC incidence. The study found that consumption of processed foods, hydrogenated oils, certain types of fish and meat, and specific cooking practices were linked to increased GBC risk. Additionally, low water intake, excessive salt consumption, and habits such as tobacco use and alcohol consumption were identified as potential risk factors. Conversely, the research suggested that some dietary choices, including regular consumption of certain oils, dairy products, and specific fruits and vegetables, might offer protective benefits against GBC. The study also highlighted the potential protective role of green tea consumption and frequent fish intake [58].

These findings contribute to our understanding of the complex relationship between diet, lifestyle, and cancer risk, emphasizing the need for further investigation into these associations to provide insights into preventive strategies and public health interventions.

Patient and Institutional Delays

A mixed-methods study at a tertiary cancer center in northeast India examined 269 breast cancer patients, revealing a median total delay of 203 days, with 35 days attributed to presentation delay and 130 days to treatment delay. Initially, 70.6% of patients sought care in the private sector, with about half consulting one healthcare provider before reaching the BBCI. Presentation delays were caused by misunderstandings of the disease, stigma, fear, symptom misinterpretation, family obligations, and discomfort with male clinicians. Treatment delays were due to underestimating disease severity, dissatisfaction with public healthcare, limited access and affordability, and fear of treatment side effects [59].

Delays in cancer diagnosis and treatment are common and linked to poor survival. A study by Bhatia et al. surveyed 214 cancer patients, with a median diagnosis age of 46 years and cervical cancer being the most prevalent (42.2%). The majority were women (81%), HIV-infected (60.7%), and had advanced cancer (56.6%). Delays were observed in appraisal (26%), help-seeking (35.5%), diagnosis (63.1%), and treatment (50.4%). Larger families reduced help-seeking delays, while women and those with severe symptoms reduced appraisal delays. Cancer type and male sex were key predictors of delays, rather than socioeconomic factors, even in a setting with free care [60].

Delayed diagnosis of cancer during patient and primary care intervals is complex and multifactorial. A systematic review by Williams et al. identified factors contributing to delayed diagnosis of gynecological cancers. These factors were further categorized into patient factors (demographics, symptoms, knowledge, presentation to general practitioners), primary care factors (doctor demographics, symptoms, knowledge, referral process), and system factors (limited access to investigations) [61].

## Conclusions

The history of cancer reflects humanity's relentless battle against a formidable disease, evolving from ancient Egyptian references to modern oncology's sophisticated techniques. Despite significant advancements, cancer remains a substantial burden, especially in developing regions like India, due to limited healthcare infrastructure, socioeconomic disparities, and cultural stigmas. Addressing risk factors like tobacco use and environmental pollutants, along with enhancing early detection and treatment accessibility, is crucial. By leveraging historical insights and contemporary innovations, we can aspire to a future where cancer's impact is significantly reduced, continuing the fight through sustained research, policy-making, and public health initiatives.
